# Recent advances in biomarkers for efficacy assessment in allergen-specific immunotherapy

**DOI:** 10.3389/falgy.2026.1793591

**Published:** 2026-06-04

**Authors:** Hong Huang, Dan Zeng

**Affiliations:** 1 Department of Pediatric, Chongqing Academy of Medical Sciences, Chongqing General Hospital, Chongqing University, Chongqing, China; 2 Department of Allergy, Chongqing Academy of Medical Sciences, Chongqing General Hospital, Chongqing University, Chongqing, China

**Keywords:** allergen, allergen-specific immunotherapy, biological markers, efficacy, therapy

## Abstract

Allergen-specific immunotherapy (AIT) is a disease-modifying therapeutic approach that addresses the fundamental etiology of allergic diseases by inducing immune tolerance through modulation of the immune system. Nevertheless, assessing AIT efficacy necessitates long-term treatment, underscoring the need for reliable predictive biomarkers. This review summarizes the recent advances in biomarker research for predicting the effectiveness of AIT, including immunoglobulins, cellular parameters, functional cytokine assays, and provocation tests. This study aims to predict the efficacy of AIT treatment early by classifying and evaluating biomarkers. Combining immunoglobulins, such as sIgE and tIgE, and their ratios, along with cytokine levels (e.g., IL-10, IL-35), could be used to predict clinical efficacy and to construct a composite prediction scoring system for improved accuracy. Monitoring of changes in sIgE, sIgG4, sIgG2, cellular markers, and cellular functions (e.g., BAT, ECP, cytokines) should be implemented to assess patient adherence and guide therapy, as they are closely associated with clinical outcomes. Although multiple biomarkers show promising potential, a standardized, unified panel of biological parameters has not yet been established. Future research should integrate multi-omics technologies, combine provocation tests with clinical evaluations, and develop accurate predictive models and more reliable, stable biomarkers for the evaluation of AIT efficacy.

## 1 Introduction

Allergic diseases pose a global health concern, particularly in more developed nations. The results of this meta-analysis showed that the total incidence of allergic rhinitis (AR) among individuals under 18 years of age in China is 17%, with a higher prevalence in women than in men. When analyzed by age group, a negative correlation is found between allergic diseases and age.

Regarding geographical distribution, the prevalence of allergic diseases among urban residents is slightly higher than among rural residents (1). A high prevalence of AR has also been recorded in developed nations, with rates in the United States ranging between 11.9% and 30.2% (2) and increasing annually (3), while dust mites remain a significant allergen. Currently, in addition to China and the United States, regions such as Europe and East Asia have also witnessed a surge in case studies on allergic diseases driven by regional environmental characteristics. The impact of dust mite allergy may continue to intensify over time (4–6).

An allergic reaction is an over-response of the immune system to a harmless substance and involves intricate immunopathologic mechanisms. When individuals with allergies are initially exposed to diverse allergens (such as dust mites, pollen, and food), antigen-presenting cells (predominantly dendritic cells) convey the allergen information to T lymphocytes. This prompts T cells to transform into a Th2-type immune response and to produce cytokines, including IL-4, IL-5, and IL-13. Cytokines IL-4 and IL-13 bind to Th2 cells and B cells. These cytokines induce a phenotypic switch in B cells, leading to the production of a substantial quantity of allergen-specific IgE (sIgE). sIgE binds to the surface of mast cells and basophils via high-affinity receptors (FcεRI), thereby causing the body to enter a sensitized state (7). Upon re-exposure to the identical allergen, the allergen binds to sIgE, thereby activating mast cells and basophils and prompting the release of a substantial quantity of inflammatory mediators (e.g., histamine, leukotrienes, prostaglandins, etc.). These mediators initiate an early-phase allergic reaction, characterized by vasodilation, smooth muscle contraction, increased glandular secretion, and stimulation of sensory nerves, giving rise to typical allergic symptoms such as nasal pruritus, sneezing, rhinorrhea, and wheezing. Multiple inflammatory cells (e.g., eosinophils, Th2 cells) infiltrate tissues and secrete cytokines and chemokines, leading to delayed-phase inflammatory reactions. Repeated, prolonged exposure to allergens can cause tissue damage and chronic inflammation.

**Figure 1:**
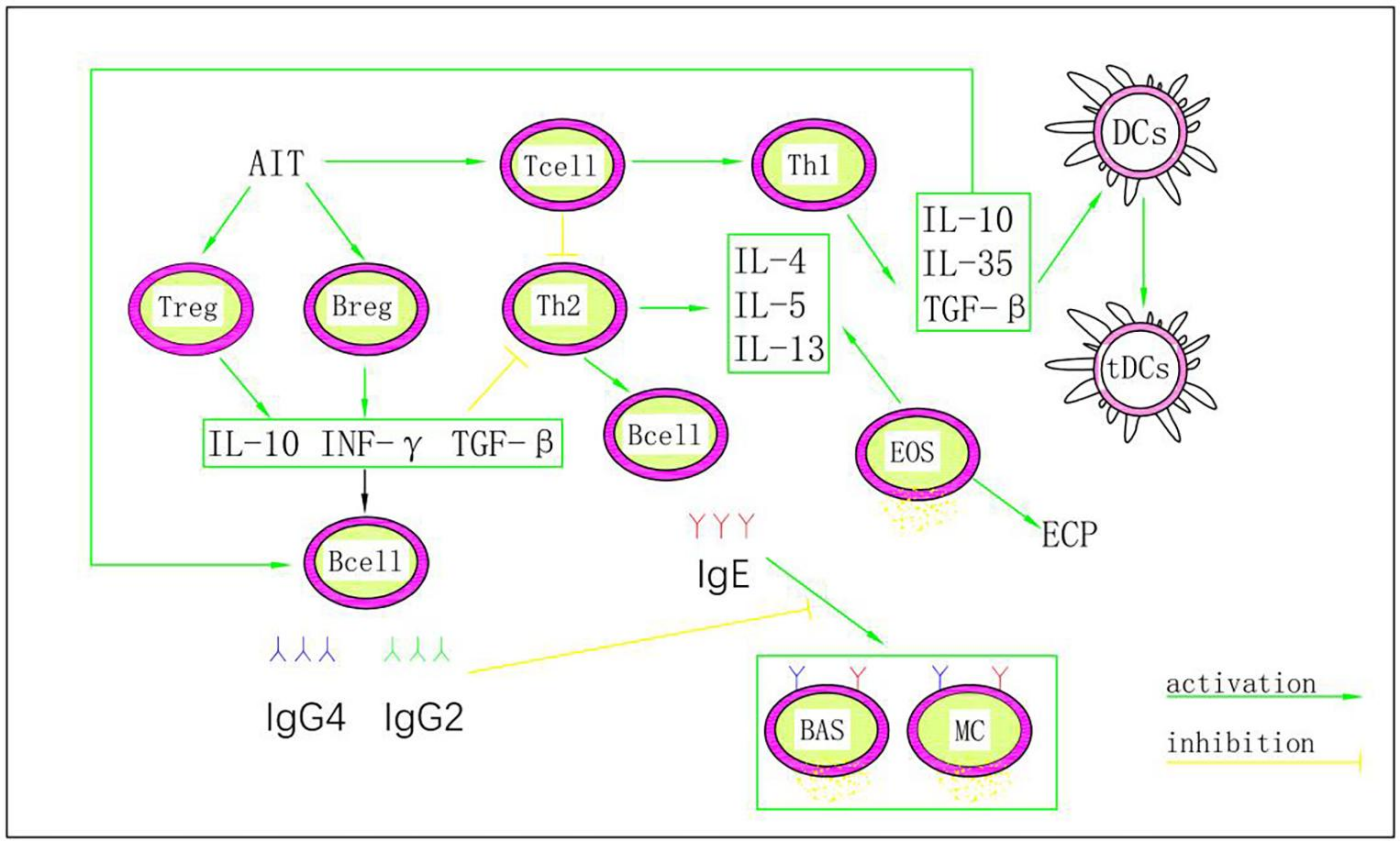
Mechanisms of AIT. AIT, allergen immunotherapy; BAS, basophils; MCs, mast cells; DCs, dendritic cells; tDCs, tolerogenic DCs; EOS, eosinophils; ECP, eosinophil cationic protein; IFN-γ, interferon-γ.

AIT, the only therapeutic approach that targets the etiology of allergic diseases, remodels the body's immune response through the gradual increase of allergen exposure and induces immune tolerance via three principal pathways (8, 9): (1) modulation of antibody levels, induction of the production of blocking IgA, IgD, and IgG antibodies (particularly the IgG4 subclass), and competitive inhibition of the binding of allergens to IgE (10); (2) effector cell desensitization by decreasing the reactivity of mast cells and basophils to allergens and diminishing mediator release; 3. Immunocyte phenotypic transformation by stimulating and facilitating the differentiation of regulatory T cells (Treg) and regulatory B cells (Breg), which secrete inhibitory cytokines, such as IL-10, INF-γ, and TGF-β, thereby preventing the activation of Th2, inhibiting the activation of relevant factors and IgE-mediated antibody responses (11), and inducing changes in dendritic cell (DCs) transformation. The mechanism of AIT is illustrated in Figure 1.

Currently, AIT modalities include subcutaneous immunotherapy (SCIT), sublingual immunotherapy (SLIT), oral immunotherapy (12), epicutaneous immunotherapy (13), and intralymphatic immunotherapy (14).

The objective of AIT is to enhance patients' allergic conditions and alleviate allergic symptoms. Statistically, it has been discovered that a complete course of AIT can notably decrease medication use and the incidence of severe disease, thus reducing the medical expenses associated with allergic diseases and improving quality of life (15). Typically, a complete course of AIT lasts longer than 3 years (16, 17). The primary efficacy endpoints include the Combined Symptom and Medication Score and the total Visual Analogue Scale. Long-term symptomatic relief and disease improvement can be achieved in approximately 65%–90% of patients (18). However, a small subset of patients with allergic rhinitis and asthma do not show a favorable response to AIT. Consequently, the exploration of biomarkers capable of accurately predicting and evaluating the efficacy of AIT is crucial for achieving individualized and precise treatment. At present, biomarkers such as immunoglobulins, cell counts, cytokines, and stimulation tests are employed in clinical practice to assess and monitor the efficacy of AIT (Tables 1, 2).

**Table 1:** Baseline biomarkers for predicting AIT efficacy.

Biological markers	Baseline stage	Prediction of AIT efficacy
sIgE/tIgE	>16.2%	Associated with successful AIT (8)
sIgE/tIgE	≤6%	Associated with unsuccessful AIT (23)
tIgE	>965 kU/L	Associated with unsuccessful AIT (23)
Pn-sIgE	≥212.3 kUA/L	Associated with successful AIT (31)
IL-10	≥98.4 pg/mL	Associated with successful AIT (21)
IL-35	≥195.2 pg/mL	Associated with successful AIT (21)
Pn-SPT	≥16.6 mm	Associated with successful AIT (31)

**Table 2:** Dynamic biomarkers for monitoring AIT.

Biological markers	Early stage of AIT	During the AIT	After AIT	Association with effectiveness of AIT
sIgE	Increased	Declined	Low level	Associated with successful AIT (18, 19, 31)
tIgE	Unchanged/increased	Unchanged/declined	Unchanged/declined	No association with successful AIT (18, 19)
sIgE/tIgE	Declined	Declined	Declined	Associated with successful AIT (18)
IgG4	Increased	High level	High level	Associated with successful AIT (19, 31)
sIgE/sIgG4	Declined	Declined	Declined	Associated with successful AIT (19, 26, 31)
IgG2	Increased	Increased	Increased	Associated with successful AIT (20)
Mast cells	Increased	Declined	Unchanged/declined	No association with successful AIT (30, 32)
Basophile	Increased	Declined	Unchanged/declined	No association with successful AIT (30, 32)
BAT	Declined	Declined	Declined	Associated with successful AIT (19, 31)
EOS	Declined	Declined	Declined	Associated with successful AIT (8, 18, 32)
Nasal EOS	Declined	Declined	Declined	Associated with successful AIT (32, 39)
ECP	—	—	Declined	Associated with successful AIT (43)
Treg (CD4 + CD25 + FoxP3 +)	Increased	Increased	High level/dropped to baseline levels	Associated with successful AIT (20)
IgE memory B cells	Unchanged	Unchanged	Unchanged	No association with successful AIT (20)
IgG2 memory B cells	Increased	Increased	Increased	Associated with successful AIT (20)
IgG4 memory B cells	Increased	Increased	Increased	Associated with successful AIT (20)
IL-4	Declined	Declined	Declined	Associated with successful AIT (18, 31)
IL-5	Declined	Declined	Declined	Associated with successful AIT (20, 31)
IL-10	Declined	Increased	Increased	Associated with successful AIT (20, 31)
IL-13	Declined	Declined	Declined	Associated with successful AIT (20, 31)
TNF-α	Unchanged/increased	Unchanged/increased	Unchanged/increased	No association with successful AIT (31, 47, 48)
IFN-γ	Unchanged/decline/increased	Increased	Increased	No association with successful AIT (20, 31, 48)
IgE-BF	Increased	High level	Slightly lower, but still higher than the baseline	Associated with successful AIT (19, 32)
IgE-FAB	Declined	Declined	Slightly higher, but still below the baseline	Associated with successful AIT (19, 32)
SPT	Decreased	Decreased	Decreased	Associated with successful AIT (20, 31, 32)
FeNO	Decreased	Decreased	Decreased	Associated with successful AIT (20)

AIT, allergen immunotherapy; BAT, basophil activation test; ECP, eosinophil cationic protein; IFN-γ, interferon-γ; IgE-BF, IgE-blocking factors; IgE-FAB, IgE-facilitated allergen binding; SPT, skin prick test; FeNO, fractional exhaled nitric oxide; —, no data available.

## 2 Immunoglobulin indexes and their clinical significance

### 2.1 sIgE and total IgE (tIgE)

sIgE is a core indicator for diagnosing allergic diseases and for determining eligibility for AIT. Transient elevations in sIgE levels are often observed during the initial phase of AIT, followed by a gradual decline (19). However, the correlation between changes in absolute sIgE concentrations and improvement in clinical symptoms remains inconsistent.

The pattern of tIgE changes during AIT remains controversial. Most studies have shown that tIgE concentrations do not change significantly during treatment, although a slight decrease, or perhaps even maintenance, may be observed during long-term follow-up (20). The tIgE levels are influenced by a variety of factors, such as age, parasitic infections, and other immune status, and their value as an indicator of AIT efficacy alone is limited. Studies have demonstrated that the more homogeneous the allergen, the closer the allergen components of AIT are to the clinical sensitizer, and the better the efficacy of AIT usually is (21).

Notably, the sIgE/tIgE ratio has emerged as an important predictor of AIT efficacy, and an analysis of AIT treatment outcomes showed that (23) low serum tIgE levels, high serum sIgE levels, and high serum sIgE/tIgE ratios were associated with better treatment responses. This finding is consistent with the recommendation of the European Academy of Allergy and Clinical Immunology, which states that an sIgE/tIgE ratio >16.2%, a sensitivity of 97.2%, and a specificity of 88.1% are good predictors of AIT clinical efficacy (8, 22). A randomized controlled open-label study of limited size could not replicate these results, while other studies showed a similar correlation between the IgE ratio and the clinical outcome of AIT. The advantage of this ratio is that it is relatively stable, reflects the intensity of sensitization by specific allergens, and is not affected by fluctuations in tIgE. Li’s study analysis (23) suggests that tsIgE > 965 kU/L and sIgE/tIgE ratio ≤ 6% were associated with an ineffective clinical response to AIT.

### 2.2 Analysis of specific IgG4, IgG2, and their ratios

Specific IgG4 (sIgG4), the principal blocking antibody induced by AIT, has been demonstrated in numerous studies to be closely associated with clinical efficacy. Throughout AIT treatment, serum sIgG4 levels can increase several-fold to several hundred-fold, a phenomenon observed in both SCIT and SLIT. By binding to allergens, IgG4 competitively inhibits the interaction between sIgE and allergen epitopes while also suppressing the formation of inflammatory complexes. Furthermore, IgG4 exhibits a half-antibody property that enables Fab-arm exchange and the formation of bispecific antibodies, thereby interfering with immune complex cross-linking (24).

In pollen SLIT, serum levels of specific IgA and IgG4 were consistently elevated, while IgE affinity was significantly reduced. The efficacy of AIT treatment was accompanied by an increase in IgG2 levels (25). Studies have also revealed that IgG2 can suppress IgE-mediated immune responses (20). This finding is particularly important because both the quantity and affinity of IgE directly influence the intensity of mast cell activation, and high-affinity IgE induces a stronger mast cell degranulation response. It has been shown that the number of Tfh13 cells, a subset of follicular helper T cells that plays a key role in the production of high-affinity IgE, reduces after SLIT, which may be responsible for the reduced affinity of IgE (24). Asthma-specific IgG2 may enhance the efficacy of immunotherapy through the following mechanisms: IgG2 has been shown to inhibit histamine release from basophils by activating FcγRIIb, potentially alleviating allergic symptoms. In addition, it promotes the expansion of memory B cells, particularly IgG4-expressing IgG+ memory B cells (CD19 + CD38dim), while IgE-expressing memory cells show no significant changes (20). Beyond these mechanisms, other IgG2-mediated allergic pathways may remain unexplored. The sIgE/sIgG4 ratio is widely recognized as a key indicator for evaluating the immune response to AIT, with a significant reduction observed during AIT treatment (26, 27), and is correlated with the suppression of delayed-phase skin reactions.

However, IgG4 has limitations as an independent biomarker: its concentration decreases by 90% post-treatment (although it remains above baseline). Also, changes in its concentration have not been shown to correlate with long-term clinical outcomes in some studies. Moreover, no correlation has been found between elevated IgG4 levels and clinical efficacy. Therefore, IgG4 is better suited as a biomarker of treatment adherence and immune response than as a predictor of long-term efficacy.

### 2.3 Sensitizing protein fractions

Serum SIgE is the most commonly used *in vitro* test for allergens and is an important part of diagnosing allergic diseases. Currently, most reagents for detecting allergen sIgE are based on crude extracts of natural allergens, which contain both allergen and non-allergen components. According to the frequency of IgE binding, allergen components are divided into major (>50%) and minor (<50%) allergen components (28). Dust mites are the most common inhalant allergens in China, and the dominant mite species are *Dermatophagoides pteronyssinus* (Der p) and *Dermatophagoides farinae* (Der f). Stratification of patients with dust mite allergy based on molecular sensitization profiles and molecular monitoring of AIT-induced IgG responses may enhance the efficacy of AIT (29).

## 3 Cellular and molecular markers

### 3.1 Mast cells, basophils, and their progenitors

Mast cells and basophils play a key role in the effector phase of the allergic reaction. AIT induces rapid desensitization of mast cells and basophils, rendering them hyporesponsive to allergens, despite high levels of allergenic sIgE observed at the start of treatment. However, the reduction of mast cell and basophil infiltration in tissues, as well as the reduction in mediator release, occurs in the late stages of AIT (30). Possible mechanisms include increased production of sIgG4 and elevated expression of low-affinity IgG receptors (FcγRIIa and FcγRIIb) on mast cells and basophils. In addition, IgG-mediated inhibition reduces the secretion of type 2 cytokines from mast cells and basophils (10).

Basophils play a central role as effector cells in allergic reactions. When the cross-linking of allergens and sIgE on the surface of basophils leads to cell activation, a series of biochemical reactions causes basophils to undergo degranulation, releasing inflammatory mediators such as histamine and leukotrienes. The expression of cell-surface markers, such as CD63 and CD203c, is upregulated during basophil activation.

All kinds of AIT can significantly reduce basophil activity (31). Studies have found that a decrease in basophil activity after 3 weeks of SCIT would predict a better response to long-term treatment (32) and that plasma from patients receiving SLIT demonstrated a stronger inhibitory effect on basophils than plasma from patients receiving OIT (as evidenced by a more pronounced decrease in CD63 expression after allergen triggering).

The basophil activation test (BAT) quantitatively assesses cellular sensitivity to allergens by detecting activated markers (CD63, CD203c) via flow cytometry. By monitoring BAT after allergen exposure, this method quantitatively assesses cellular sensitivity to these triggers (19). A novel assay to assess the extent of degranulation by measuring intracellular diamine oxidase (DAO) levels found a significant decrease in DAO after AIT and a positive correlation between the decrease in DAO and the amount of histamine released (33).

However, the clinical application of BAT still faces challenges: approximately 10%–20% of patients have basophils that do not release histamine when stimulated by positive controls (e.g., anti-IgE antibodies) despite responding normally to non-specific stimuli. This phenomenon may be related to differences in the intracellular signaling pathway of the high-affinity IgE receptor, FcεRI. Although these patients exhibit defective basophilic responses, they can still mediate the inflammatory response via mast cells. However, there is no reliable method to identify such “non-responders” in advance, highlighting the need for a standardized BAT procedure (34). It should be emphasized that the basophil activation status monitored by BAT has been regarded as an important biological marker for the establishment of immune tolerance (27).

Mast cell progenitors (MCPs) are derived from differentiated hematopoietic stem cells in the bone marrow or spleen and enter the vascular circulation. A 2023 study on birch pollen-allergic asthma (35) found that the number of MCPs in the peripheral blood of patients increased significantly during the pollen season and that sustained activation of mast cells depended on the differentiation and proliferation of MCPs, with the quantity of MCPs showing a negative correlation with asthma symptoms and control scores. This finding reveals the dynamic effects of allergen exposure on the mast cell lineage and suggests that MCPs may serve as a novel biomarker for assessing AIT efficacy (36).

### 3.2 Eosinophils, eosinophil chemokines, and eosinophil cationic protein

Eosinophils (EOS) promote inflammatory response and tissue damage by releasing various inflammatory factors and cytokines (e.g., IL-5, IL-4, and IL-13, etc.) and contribute to the assessment of AIT efficacy (32), with lower levels of EOS being associated with better treatment outcomes than higher EOS levels (8). However, some statistical analyses have shown that blood EOS levels are not significantly associated with clinical efficacy after AIT treatment (23).

Nasal cytology, an AR important diagnostic tool, can directly identify EOS infiltration (a core marker of allergic inflammation) by microscopic examination of mucosal scrapings from the inferior turbinate (37). Studies have confirmed that tIgE levels are positively correlated with nasal mucosal eosinophilia (38). For the management of patients with rhinitis who show negative or insufficient evidence of systemic atopic markers, a therapeutic trial with nasal steroids and oral antihistamines is recommended when elevated tIgE levels are present, which can be further assisted by nasal cytology and nasal provocation test (NPT) if first-line treatment is not effective. Therefore, nasal EOS may serve as one of the indicators for predicting and monitoring the efficacy of AIT (39), especially in allergic rhinitis; however, this marker is clearly not applicable to all patients with allergic diseases.

EOS chemokines are members of the CC chemokine ligand (CCL) family, consisting of EOS eotaxin-1 (CCL11), EOS eotaxin-2 (CCL24), and EOS eotaxin-3 (CCL26), with distinct immunoreactivities. These chemokines are key mediators of EOS migration, activation, and maturation. High levels of EOS chemokines promote local and systemic EOS recruitment, thereby contributing to the pathophysiology of various autoimmune and inflammatory diseases (40). However, their specificity is low, and there is no standard threshold for predicting or evaluating AIT efficacy, which limits their clinical application.

Allergic rhinitis is a non-infectious inflammatory disease of the nasal mucosa, mainly mediated by IgE, after exposure to allergens, accompanied by the aggregation of EOS. Eosinophil cationic protein (ECP) is a granule protein released mainly by EOS during degranulation. Its turnover time in the blood is about 45 min. Therefore, ECP is recognized as an important marker of EOS activity and regression and has been widely used in the diagnosis and efficacy assessment of EOS-related diseases (e.g., allergic diseases) (41, 42). Elevated serum ECP levels during allergic episodes can be used as an auxiliary monitoring indicator during AIT. AIT could effectively reduce serumal ECP levels by reducing EOS granulocyte aggregation and activation (43). Studies have shown that ECP concentration is a potent biomarker of type 2 inflammation in children, helping identify those at the highest risk of recurrent exacerbations and guiding them toward corticosteroid therapy. In addition, ECP tends to decline rapidly than EOS over the course of disease treatment and can be used to predict the efficacy of asthma treatment (44).

### 3.3 Tregs and Bregs

Tregs and Bregs play central roles in AIT-induced immune tolerance (9). Tregs include both natural regulatory T cells (nTregs) and induced regulatory T cells (iTregs), which function through the expression of Foxp3 transcription factors and the secretion of inhibitory cytokines (IL-10, TGF-β, IL-35). Among these, Tregs expressing high levels of CD25 and FoxP3 exhibit the highest inhibitory capacity. AIT promotes the expansion of Tregs, inhibits Th2 cell activity, and re-establishes immune homeostasis. Bregs specifically produce IL-10, inhibit antigen presentation by downregulating MHC-II expression, promote IgG4 class switching, and inhibit IL-4-induced IgE synthesis. AIT induces Bregs to produce high levels of IL-10, which effectively inhibits antigen-specific CD4+ T cell proliferation (26) and also suppresses Th2 cell differentiation and effector functions, thereby reducing allergic inflammation. However, there are significant challenges to the clinical application of cytologic parameters, including the scarce numbers of Tregs and Bregs, the lack of specific surface markers for quantitative detection (reliance on functional assays), and changes in the numbers that precede clinical symptom improvement, with a weak correlation with short-term symptomatic improvement. Although these cytological parameters are currently theoretically promising, they still require further academic research support and are not yet applicable in clinical practice.

### 3.4 Dendritic cells (DCs)

DCs, as key antigen-presenting cells, play an important role in early immune remodeling in AIT. Microenvironmental factors tightly regulate the direction of differentiation during antigen uptake of immature DCs. In the absence of inflammation during antigen uptake, the presence of inhibitory cytokines such as IL-10 and TGF-β and microenvironmental triggering molecules such as TLR2, TLR7, or TLR9 can induce the transformation of immature DCs into tolerogenic DCs (tDCs), which exhibit low expression of CD80, CD86, and MHC-II. tDCs can prevent allergic inflammation (45). Studies have shown that SLIT can significantly affect the number of blood DCs, upregulate DCreg markers (e.g., C1q), and downregulate DC2-associated molecules. AIT-induced DCs not only support the differentiation of Tregs but also mediate anti-inflammatory effects directly, which is a potential indicator of early therapeutic efficacy. DCs exhibit large heterogeneity in cell subpopulations, and isolating and culturing them is costly and difficult to implement in the clinic; however, they can be considered as adjuvants for AIT treatment to enhance its effectiveness.

## 4 Cytokine, IgE-BF, and IgE-FAB

### 4.1 Cytokines

AIT can reshape the cytokine profile in allergic patients, shifting from a Th2-dominant response (characterized by IL-4, IL-5, and IL-13) toward Th1-mediated (e.g., IFN-γ) and regulatory (e.g., IL-10, TGF-β) responses.

In a study of SLIT in patients with peanut allergy (31), peanut stimulation led to significantly reduced levels of Th2 cytokines (IL-4, IL-5, IL-13) after 12 months of treatment compared with baseline, and this reduction persisted after SLIT completion. In contrast, IFN-γ levels decreased significantly only after 48 months of therapy, while TNF-α showed no notable change throughout the SLIT process.

Another study involving patients with dust mite-induced allergic rhinitis identified cytokines such as IL-4, eotaxin, and IFN-γ as potential early biomarkers for predicting the efficacy of SCIT in children. Specifically, low IFN-γ and high IL-4 levels were found to be more reliable predictors of AIT outcomes, with serum IL-4 demonstrating superior predictive reliability compared to eotaxin and IFN-γ (46).

However, some studies have also reported an increase in IL-5 levels following peanut OIT. Conversely, IFN-γ and TNF-α have shown divergent expression patterns during AIT: in some cases, their levels increased significantly as early as several months after treatment initiation (47), while in others, a gradual decrease was observed during the course of therapy (48).

IL-10 is a key regulator of inflammatory responses, protecting the host from tissue damage caused by excessive inflammation. It inhibits antigen presentation and the production of pro-inflammatory chemokines and cytokines. SLIT can induce IL-10 and IL-35, which can exert protective effects. Therefore, higher baseline levels of IL-10 and IL-35 may predict a good response to SLIT (21). Liu suggested that SLIT predicts the thresholds for efficacy as serum IL-10 ≥ 98.4 pg/mL (sensitivity 84.5%, specificity 72.1%) and serum IL-35 ≥ 195.2 pg/mL (sensitivity 88.9%, specificity 70.6%). In patients with rhinitis or asthma, analyzing cytokines in local samples, such as nasal secretions or bronchoalveolar lavage fluid, may be more valuable. Notably, following allergen challenge, AIT-treated patients demonstrate significantly reduced concentrations of Th2 cytokines and chemokines in nasal secretions.

However, the clinical utility of serum cytokine measurements remains limited because of multiple confounding factors and their poor correlation with clinical efficacy. Key limitations include their low circulating concentrations and short half-lives, which require highly sensitive detection techniques; the poor ability of systemic levels to reflect local tissue inflammation; and the lack of a consistent association with symptom scores.

### 4.2 IgE-blocking factor (IgE-BF) and IgE-facilitated allergen binding (IgE-FAB)

IgE-BF and IgE-FAB are two indicators used to monitor the suppression of allergic reactions and quantify the effectiveness of AIT in suppressing allergic reactions. These markers also represent potential directions for future research. IgE-BF reflects the extent to which factors such as serum IgG4 prevent the binding of sIgE to allergens. IgE-FAB indicates the binding of allergen–IgE complexes to FceRII (CD23) on the surface of B cells, a critical step in subsequent IgE-mediated antigen presentation. Studies have shown that IgE-BF levels after 4 months of treatment can predict efficacy after 1 year of treatment (49). Statistical analysis has revealed that AIT-induced reductions in IgE-FAB and their association with clinical outcomes. Therefore, IgE-BF and IgE-FAB deserve further investigation as biomarkers for predicting and monitoring AIT efficacy (19).

## 5 Excitation test

### 5.1 Skin prick test (SPT)

The SPT is a provocative test that visualizes the reaction of the body by introducing an allergen into the skin. A study by Kim et al. on peanut allergy (31) showed that patients were less likely to naturally outgrow their peanut allergy. Baseline biomarkers indicated that the participants were unlikely to naturally outgrow their peanut allergy with a baseline Pn-SPT of 16.6 mm and a Pn-sIgE level of 212.3 kUA/L. SPT and BATs decreased after peanut SLIT. SPT has been clearly shown (20) that the diameter of wheals post-SLIT procedure is positively correlated with the severity of clinical symptoms of allergic diseases, and a reduction in diameter indicates improvement of allergic symptoms.

### 5.2 Nasal provocation test (NPT)

The NPT is assessed by measuring nasal symptom scores and nasal airway resistance before and after allergen provocation. Studies have demonstrated correlations between NPT responses, the size of SPT air masses, and serum sIgE levels; however, only the intensity of the NPT response has been shown to significantly correlate with the severity of the actual nasal symptoms of the patient (50). NPT can be used not only for inhalant allergens but also for screening for food allergies. In addition, it can be used in conjunction with exhaled nitric oxide (FeNO) testing to assess the provocation responses, assist in the diagnosis of allergy, and monitor the efficacy of AIT (37, 51). However, NPT has significant limitations: viral infections can induce the release of histamine and pro-inflammatory mediators in nasal secretions, which can interfere with the results. Therefore, NPT should be performed at least 2–4 weeks after the resolution of any acute allergic attack or respiratory infection (52).

All provocative tests carry the risk of inducing allergic reactions. First-aid medications and equipment must be available in the examination room, and a comprehensive first-aid plan must be in place. Given the potential risks, the clinical use of provocation tests for allergy screening and AIT efficacy monitoring should be carefully evaluated.

## 6 Discussion

Despite the potential of many biological indicators, the evaluation of AIT efficacy faces multiple challenges: (1) individual response heterogeneity: factors such as genetic background, type of allergen, clinical manifestations, allergic exposure pathways, and AIT regimen (dose, route, and duration of treatment) significantly affect the pattern of immune response; (2) complexity of dynamic changes: different indicators show non-linear patterns of changes over time (e.g., initial elevation of sIgE); (3) differences between local and systemic immunity: changes in the local immune microenvironment of tissues are difficult to be accurately reflected by peripheral blood tests; (4) lack of methodological standardization, such as the setting of the CD63 threshold in BAT and the difference in sensitivity of cytokine detection; and (5) issues such as the safety of the excitation test.

To address the aforementioned challenges, future efforts could consider developing integrated multi-index models and establishing individualized efficacy evaluation and monitoring standards for different allergen types and AIT methods. Given the limited predictive value of single biomarkers, constructing a composite prediction scoring system may enhance predictive accuracy. For instance, in the presence of clinical symptoms or positive provocation test results, combining immunoglobulins (sIgE and tIgE) and their ratios, as well as cytokine levels (e.g., IL-10, IL-35), could be used to predict clinical efficacy and to construct a composite prediction scoring system for improved accuracy. During AIT treatment, regular monitoring of changes in sIgE, sIgG4, sIgG2, cellular markers, and cellular functions (e.g., BAT, ECP, cytokines) should be performed to assess patient adherence and guide therapy. Concurrently, there is an urgent need to develop more novel, safe, and standardized provocation tests and monitoring methods.

Long-term observational studies have provided strong evidence for the disease-modifying effects of AIT. A 20-year follow-up study by Magnus and colleagues involving 1,137 pollen-allergic individuals showed that 75% of those who developed allergic rhinitis due to pollen allergy during childhood continued to experience symptoms into adulthood, and 30% eventually progressed to asthma (53). A follow-up study of patients with dust mite allergy for up to 15 years found that the clinical benefit persisted for up to 7 years after 3 years of SLIT treatment and for up to 8 years after 4–5 years of SLIT treatment, suggesting that AIT significantly reduces the risk of disease persistence (54). This long-term protective effect is associated with the formation of immune memory, but specific biological indicators reflecting this effect still need to be clarified, and there is a lack of research on and established criteria for a standardized treatment course.

In addition to pure AIT therapy, the combination of biologics and adjuvants can improve AIT efficacy to some extent across various clinical scenarios. By combining the disease-modifying effects of AIT with the targeted therapeutic actions of biologics, biologics demonstrate enhanced efficacy. For example, the combination of Dupilumab with mite allergen-specific immunotherapy has demonstrated significant clinical improvement in children with moderate-to-severe atopic dermatitis, without any new adverse reactions (55). Omalizumab in combination with AIT therapy can effectively enhance asthma control and improve pulmonary function (56). Adjuvants interact with antigens through physical or chemical mechanisms, thereby altering the pharmacological and immunological effects of allergens. Examples include aluminum hydroxide, microcrystalline tyrosine, calcium phosphate, monophosphoryl lipid A, toll-like receptor agonists, liposomes, and others. Nagy (57) suggested that vitamin D may potentially serve as an adjuvant in the regulation of allergic diseases in the future.

With the development of gene technology, more and more allergen-susceptibility loci have been identified. In the future, it may be possible to assist in the clarifying allergens by analyzing specific susceptibility gene profiles and to explore the use of gene technology to monitor the efficacy of AIT. Concurrently, precision therapy using genetic technologies has the potential to modify human allergic responses, thereby addressing the root causes of allergic diseases.

## 7 Conclusion

The investigation of biomarkers is of substantial significance for comprehending the mechanisms of AIT and for refining clinical practice. Conventional indicators, including serum immunoglobulin levels (such as sIgE, tIgE, and sIgG4), eosinophil-related parameters, the activation status of mast cells and basophils, and ratios such as sIgE/tIgE and sIgE/sIgG4, have shown considerable value in predicting and monitoring the efficacy of AIT. Simultaneously, emerging biomarkers—such as major allergen-specific IgE and ECP as potential early predictors, along with elevated levels of sIgG2 and sIgG4, their ratios to sIgE, basophil activity, nasal EOS counts, IgE-BF, and IgE-FAB—offer new perspectives and understandings for treatment evaluation. However, clinical decision-making should continue to prioritize symptom control and improvement in quality of life as the primary endpoints.

Future research should concentrate on the following aspects: formulating standardized detection protocols based on biomarker research; clarifying the relationship between changes in the local immune microenvironment of tissues and peripheral biomarkers; establishing integrated multi-parameter predictive models to enhance the accuracy of efficacy prediction; and exploring the role of novel indicators, such as epigenetic markers, in AIT response and long-term outcomes. By promoting biomarker-guided personalized AIT strategies, it is expected that treatment response rates will be significantly enhanced, therapy course management will be optimized, and the ultimate objective of precision immunotherapy for allergic diseases will be achieved.
